# Competing Self-Trapped Exciton States and Multiple Emission Pathways in BiVO_4_

**DOI:** 10.1021/acs.jpclett.5c01215

**Published:** 2025-06-26

**Authors:** Tobias Möslinger, Nicklas Österbacka, Julia Wiktor

**Affiliations:** 11248 Chalmers University of Technology, Department of Physics, 41296 Gothenburg, Sweden

## Abstract

Transition metal oxides, such as BiVO_4_, have attracted significant attention for their potential in photoelectrochemical water-splitting. BiVO_4_, a model material in this area, is prone to charge localization in the form of small polarons. Recently, self-trapped excitons (STEs) in BiVO_4_ have been experimentally observed, but their precise nature remains elusive. In this study, we employ time-dependent density functional theory (TD-DFT) with a nonempirical PBE0­(α) hybrid functional to investigate the localization, stability, and optical properties of STEs in BiVO_4_. Our results reveal two distinct localized exciton configurations with comparable energies. We show that the emission from a single STE configuration leads to multiple peaks in the emission spectrum, originating from different types of internal transitions. The positions of peaks in the calculated optical spectra are in good agreement with experimental observations.

## Graphical abstract

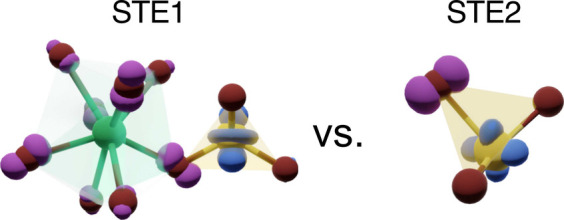

Transition metal oxides have emerged as a promising class of materials for photoelectrochemical water-splitting, which is a reaction that transforms solar light into hydrogen and oxygen. Among several promising materials, BiVO_4_ is one of the most studied and has become a platform for understanding phenomena occurring in a larger class of materials. One interesting property of BiVO_4_ is that the excess charges have the tendency to localize in this material.^1–15^

When BiVO_4_ absorbs a photon, an electron–hole pair is created. Often, the two excess charges are considered independent, however, in a real material they will interact with each other, forming excitons. Excitonic effects in BiVO_4_ have been shown to be significant even at room temperature, with an estimated exciton binding energy of 0.11 eV.^16^ Considering both significant excitonic effects and the tendency of BiVO_4_ to trap charges, one can also expect the appearance of self-trapped excitons (STEs) in the material. Indeed, these quasiparticles have become of interest in bismuth vanadate, with several experimental reports published recently.^15,17–19^ While the experimental evidence for the presence of STEs is clear, it is not straightforward to extract the exact nature of these states from measurements. Therefore, it is of interest to investigate the localization of electron–hole pairs in the materials computationally as well.

Modeling STEs poses two challenges. First, one needs to use methods able to describe the excited state, which go beyond the standard density functional theory (DFT). This can be achieved by solving the Bethe-Salpether equation, as has been done for example for defected surfaces of BiVO_4_ by Steinitz-Eliyahu et al.^20^ Alternatively, advances in the accuracy of methods based on time-dependent density functional theory (TD-DFT), in particular coupled with nonempirical hybrid density functionals,^21–25^ allow for comparable results at a lower computational cost. Time-dependent DFT coupled with hybrid functionals has been recently applied to study STEs in perovskite semiconductors.^26,27^ Second, the charge localization needs to also be treated correctly. This means that the self-interaction error that is present in the standard semilocal DFT has to be corrected. This can also be achieved by the use of nonempirical hybrid functionals in which the fraction of exact exchange is chosen to remove the aforementioned error.

In the present study we apply the TD-DFT method coupled with the PBE0­(α) hybrid functional to explore the nature, stability, and emission of STEs in BiVO_4_. We find two possible configurations of the localized exciton, with similar energies. We find that both configurations lead to multiple peaks in the emission spectra. This implies that multiple photoluminescence peaks observed recently in experiments^18^ potentially originate from different transitions within a single STE, rather than from distinct charge-localized states.

We first determine the α parameter within the PBE0­(α) hybrid functional that removes the self-interaction error, by verifying that the Koopmans’ condition is fulfilled.^28–30^ To this end, we use the cp2k code^31,32^ to calculate the single-particle levels of the electron polaron in BiVO_4_ with different values of α and apply the correction scheme of Falletta et al.^33^ We consider the dielectric constants *ε*_*∞*_ = 5.83 and *ε*_0_ = 64.95.^34^ For computational convenience, we use a 2 × 2 × 2 repetition of the experimental tetragonal scheelite structure (*a* = *b* = 10.294 Å, *c* = 23.443 Å) as determined by Sleight et al.,^35^ containing 192 atoms. This phase exhibits electronic properties very similar to those of the experimentally relevant monoclinic scheelite form.^6,11^ We compared the properties of one of the STE configurations between the tetragonal and monoclinic models (see Figure S5) and found no significant differences. The appropriate α value can be determined as the intersection of the straight-line fits to the occupied and unoccupied polaron states.^34^ A value of 0.14 fulfills that condition, as seen in Figure 1. This is in agreement with the result of Faletta and Pasquarello.^34^ As shown in the Supporting Information (SI), this result is additionally insensitive to the choice of supercell size.

**Figure 1:**
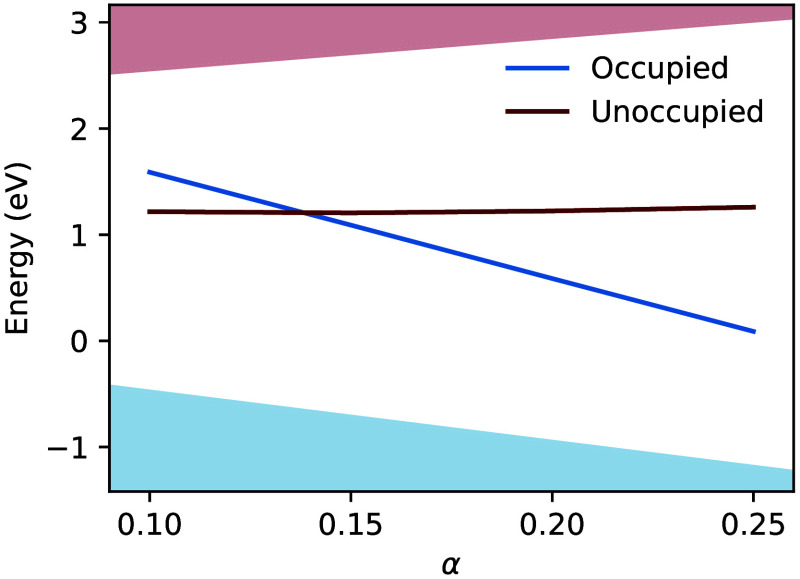
Electron polaron single-particle levels as well as the valence band maximum and conduction band minimum as functions of exact exchange α. Energies are relative to the valence band maximum at α = 0.

We now identify the possible STE configurations, again using cp2k. First, we relax the structure of BiVO_4_ under the constraint of keeping the tetragonal scheelite symmetry. The resulting lattice parameters are *a* = *b* = 10.311 Å, *c* = 23.594 Å. We then perform calculations in the triplet state, which results in the excitation of an electron–hole pair. We introduce initial distortions around different configurations of V and Bi atoms to initiate charge localization and then relax the structure. We find two distinct configurations in which the two charges localize in close vicinity of each other. In the first (STE1), the electron localizes on a single V atom while the hole localizes onto a neighboring BiO_8_ octahedron as shown in Figure 2a. In the second (STE2), the electron also localizes on a single V atom, although in a different symmetry, while the hole localizes onto one of the neighboring oxygen atoms as shown in Figure 2b.

**Figure 2:**
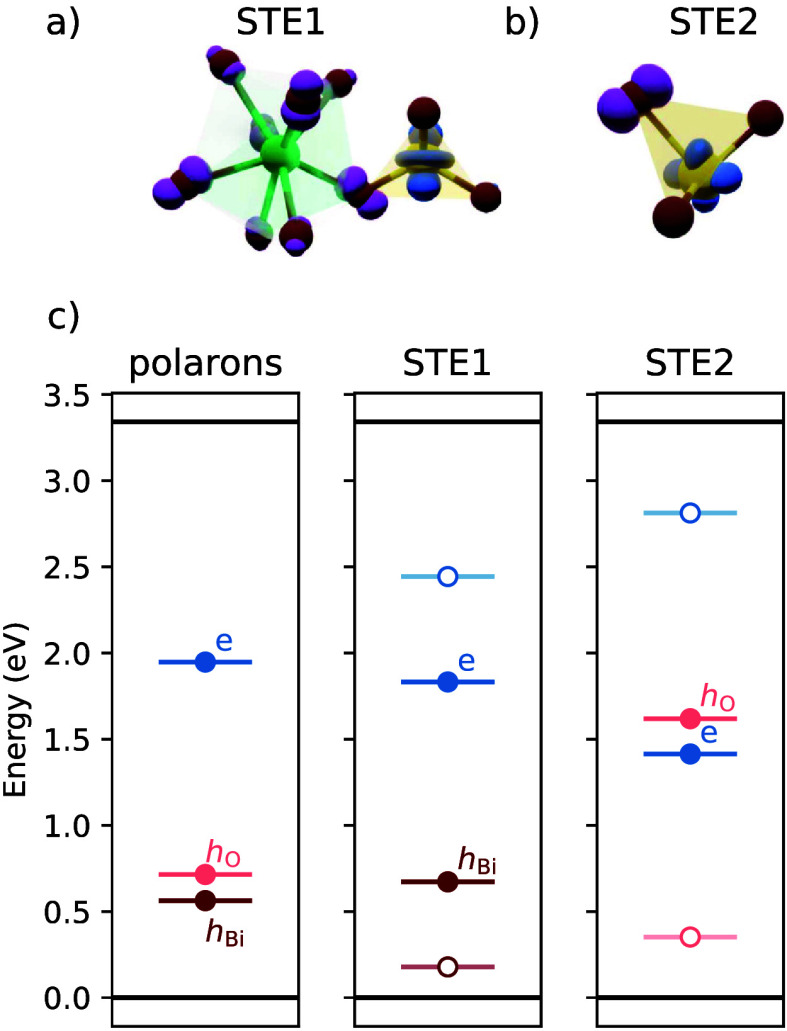
Schematic depiction of charge localization in STE1 (a) and STE2 (b), with O, Bi, and V atoms colored red, green, and yellow, respectively. The hole isodensity is shown in purple and the electron isodensity in blue. (c) Single-particle energy levels associated with the separate polaronic states and localized charges within the STEs.

We now focus on the formation energies of the STEs, *E*_f_^STE^, defined as

$$E_{f}^{\mathrm{STE}}=E_{\mathrm{STE}}-E_{\mathrm{pristine}}+\epsilon_{\mathrm{VBM}}-\epsilon_{\mathrm{CBM}}$$ 1

where *E*_STE_ is the total energy of the cell with an STE, *E*_pristine_ the total energy of the neutral pristine system and ϵ_VBM_ and ϵ_CBM_ the energy of the valence band maximum and conduction band minimum, respectively. We note that calculations involving STEs in periodic supercells might lead to spurious dipole–dipole interactions. Additionally, *E*_f_^STE^ in BiVO_4_ may be sensitive to the *k*-space sampling density, which is limited to the Γ point in cp2k. We therefore evaluate the finite-size effects by performing calculations in different sizes of supercells. These tests are given in SI (see Figure S2). We conclude that the formation energy of STE1 calculated in the 2 × 2 × 2 supercell within cp2k is about 0.16 eV below the extrapolated value. For the more localized STE2, the discrepancy is lower and amounts to 0.07 eV only. Since the deviation for STE1 is relatively large, we decide to use a larger cell (768 atoms, 4 × 4 × 2 repetition of the unit cell) to calculate formation energies in the following. Since we are also interested in the photoluminescence spectra from the STEs, we also verify the convergence of the emission energy. This is approximated by the vertical transition energy, calculated as the energy difference between the triplet state and the ground-state singlet in the STE geometry. This difference is not necessarily the same as the TD-DFT transition energy but serves as a good predictor.^36^ Figure S3 shows that the vertical transitions are converged within 0.01 eV already in the 2 × 2 × 2 supercell. Therefore, we will use this cell in the following TD-DFT calculations.

We now take the configurations of the two STE states from cp2k and perform additional calculations within vasp.^37,38^ We first calculate formation energies for the two configurations within constrained DFT in the triplet state. We use the cell with 768 atoms coming directly from cp2k. Formation energies, calculated using eq 1, are given in Table 1. In the table, we also include the formation energies of separate polarons that constitute the STEs, calculated within the current computational setup as

$$E_{f}=E^{q}\left[\mathrm{pol}\right]-E^{0}\left[\mathrm{pristine}\right]-\epsilon_{ref}+E_{\mathrm{corr}}$$ 2

where *E* ^*q*^[pol] is the total energy of the relaxed supercell containing the small polaron in charge state *q* (*q* = – 1 for an electron, +1 for a hole), *E* ^0^[pristine] is the energy of pristine BiVO_4_, ϵ_ref_ is the position of the relevant band edge (CBM for electron polarons and VBM for hole polarons), and *E*_corr_ accounts for the electrostatic finite-size correction.^39^

**Table 1:** Formation Energies (*E*_f_) of Different Self-Trapped Exciton (STE) States and Separate Polarons in BiVO_4_ ^a^

	*E*_f_ (eV)	*E*_f_ (eV)
State	α = 14%	α = 22%
STE1	–0.88 (0.04)	–1.58 (0.15)
STE2	–0.85 (0.01)	–1.74 (0.31)
Hole (Bi)	–0.23	–0.35
Hole (O)	–0.10	–0.34
Electron	–0.61	–1.08

a Binding energies for the STEs, calculated relative to the most stable separate polaron configuration, are shown in parentheses. Calculations were done in the 768-atom supercell.

Within PBE0(14%), STE1 is found to be more stable than STE2 by only 0.03 eV. This small energy difference is within the accuracy of such calculations. We therefore conclude that both configurations are likely to exist in the material. To further explore STE formation from separated polarons and assess their binding energy, we recalculated the energies of isolated hole and electron polarons using the same computational framework. The results are included in Table 1. For the hole polaron, we construct two configurations corresponding to the localization found within STE1 and STE2, namely with the density on either eight oxygens surrounding one Bi atom (*h*_Bi_), or mostly on a single oxygen (*h*_O_). In the case of separate polarons, we consider finite-size corrections for both total energies and single-particle levels.^39,40^ By comparing the formation energies of the STEs with the combined formation energies for separate polarons, we find relatively weak binding energies of 10–40 meV when α = 14*%* is used. We also recalculate the formation energies for a higher value of α = 22*%*, which has been shown to reproduce the band gap calculated within the QS*GW̃* method.^16^ We find that with the increased value of α the binding energies of the STE are increased to 150–310 meV, with STE2 being more stable. The inverted order of stabilities can be related to the fact that with α = 22*%* the localization of the hole polaron onto one oxygen atom becomes relatively more stable. We note that the dependence of the relative stability of the two types of hole polarons in BiVO_4_ is in line with previous work by Liu et al.^10^ While the binding energies of hole polarons are enhanced at α = 22*%*, they remain lower than those of the electron polaron, consistent with the pronounced asymmetry in charge transport observed experimentally.^41^ The variation of STE binding energies with the hybrid functional underscores a broader challenge in modeling BiVO_4_, where different high-level methods, including Koopmans-compliant hybrids and QS*GW̃*, predict significantly different band gaps, in contrast to their close agreement for many other semiconductors.^29,30^ As no universally established parametrization exists for BiVO_4_, we employ the Koopmans-derived α in the main calculations due to its relevance for localized states, while also including results for the α that reproduces the QS*GW̃* band gap. Given the experimental observation of STEs at room temperature,^18^ the larger binding energies obtained with α = 22*%* may be more physically relevant. These findings point to the need for further theoretical and experimental studies to resolve the parametrization and provide a more robust estimate of the true STE binding energy in BiVO_4_.

We now analyze the single particle levels related to the separate polarons and STEs, as shown in Figure 2. The occupied levels within STE1 correspond well to the levels of the separate polarons (see Figure 2c). The stabilization due to electron–hole interaction moves them by 0.12 (electron) and 0.11 eV (hole) deeper into the band gap, as compared to the noninteracting states. Unlike for the separated polarons, where generalized Koopmans’ theorem holds, the levels within the STE become more shallow once the interacting charges are removed from the cell. In the case of STE2, which is a more localized state, the stabilization of the electron and hole levels is significantly stronger (0.53 eV for the electron and 1.06 eV for the hole), leading to inverted ordering of electron and hole levels within the band gap.

We now perform the TD-DFT simulations for the STEs to understand their emission properties. Calculations are done within the 2 × 2 × 2 supercell containing 192 atoms, as we have found that it is enough to obtain well-converged emission energies. We perform calculations in the ground state of STE1 and STE2 and consider transitions to different excited states. In the calculations, we include 50 occupied and 50 unoccupied states and use a 2 × 2 × 2 *k*-point mesh.

In Figure 3 we present the optical transitions from STE1 and STE2, along with their corresponding band contributions, which provide insight into the nature of the electronic states involved in each transition. We plot all transitions obtained from TD-DFT calculations, treating emission as the inverse of absorption in the self-trapped exciton (STE) geometry, without assuming a specific excited-state population distribution. The band contributions are determined by summing over the intensities of all transitions that involve a given electronic state, allowing us to assess the extent of localization vs delocalization in the excited-state dynamics. According to the Kasha rule, emission should originate only from the lowest-energy excited state.^42^ However, experimental results suggest multiple competing emission pathways,^18^ likely due to the limited mobility of localized charge carriers, which prevents full relaxation to the lowest-energy state.

**Figure 3:**
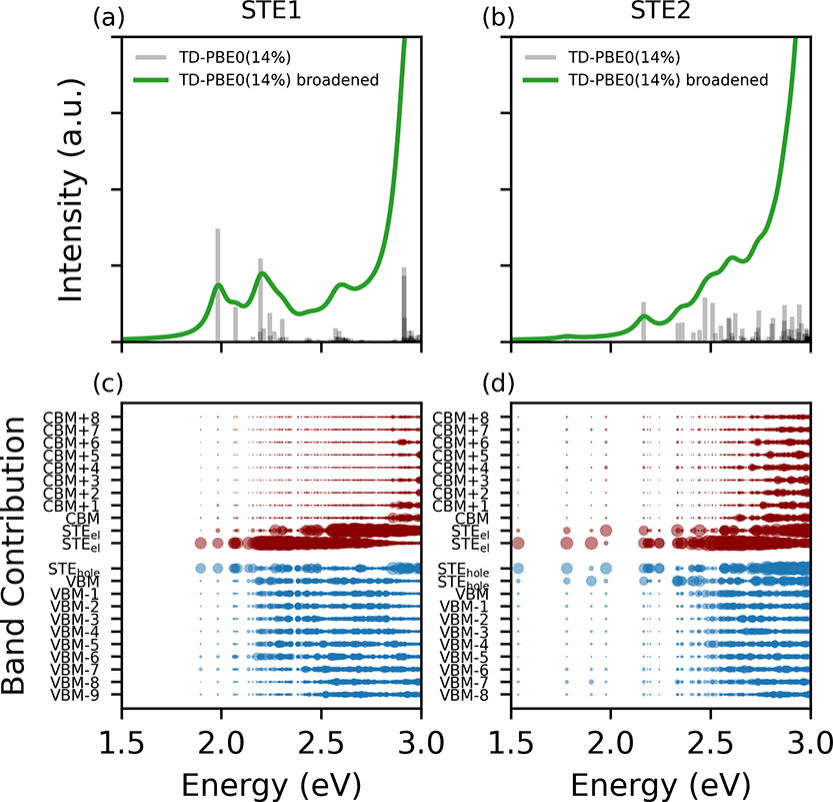
Optical transitions calculated for the STE1 (a) and STE2 (b) configurations. The broadened spectra were generated by convolution with Lorentzians with a width of 0.05 eV. (c and d) Band contributions to each of the transitions marked in the corresponding upper panel. For each initial (final) state, the contributions of different final (initial) states, as well as all *k*-points, are added up.

For STE1, we observe several distinct peaks below the sharp increase in intensity at the energy where the absorption edge of the pristine material is expected. The most pronounced peaks appear at 1.98, 2.20, and 2.60 eV, with the lowest transition at 1.90 eV being dark due to its triplet nature. The band contributions in Figure 3c suggest that the lower-energy transitions originate from recombination between localized electron and hole states within the STE, whereas the higher-energy transitions have a mixed character, also involving delocalized VBM states. The STE2 state (see Figure 3b and d) exhibits less pronounced peaks and a broader distribution of possible transitions. This suggests that its presence would be difficult to distinguish from that of STE1, although it could still contribute to the experimentally observed photoluminescence. We note that STE1 and STE2 differ in the number of localized in-gap states related to holes, with the former having only one such state and the latter exhibiting an additional excited orbital. The nature of each state, localized or delocalized, was identified by visualizing the corresponding Kohn–Sham wave functions.

The presence of multiple peaks is consistent with what was observed experimentally in ref 18, where photoluminescence measurements on BiVO_4_ thin films revealed a broad asymmetric emission band, deconvoluted into two lower-energy peaks at 1.75 and 1.95 eV, and two higher-energy peaks at 2.56 and 2.63 eV. We note that these thin-film samples were under strain, and the transition energies shifted higher as the film thickness increased.

While experimental studies attributed these peaks to distinct localized states, our calculations suggest that they can all arise within a single STE. However, a separate electron polaron would be expected to exhibit transitions similar to those found in our calculations at about 2.20 and 2.60 eV, corresponding to the recombination between the electron within the STE and VBM states, potentially contributing to the observed spectra. Although exact energy positions may differ due to computational approximations and structural factors, our results align well with experiment, particularly in the energy spacing between peaks and the presence of multiple emission pathways, whether from a single STE or different localized charge-carrier states, as seen in experiment.

In conclusion, we have investigated the nature, stability, and emission properties of self-trapped excitons (STEs) in BiVO_4_ using time-dependent density functional theory with the nonempirical PBE0­(α) hybrid functional. Our study identifies two distinct STE configurations with comparable formation energies, suggesting that both types may coexist in BiVO_4_ under experimental conditions.

We find that the emission from a single STE configuration leads to multiple peaks in the luminescence spectrum. These peaks originate from transitions involving localized charge carriers within the STE as well as from transitions involving more delocalized states. This interpretation provides an alternative explanation to recent photoluminescence measurements, which previously attributed the observed multiple emission peaks to different types of excitonic quasiparticles.

Furthermore, our calculations reveal that the binding energy of STEs in BiVO_4_ is relatively low, which suggests that thermally activated dissociation into free polarons may compete with exciton localization under ambient conditions. The energy ordering of STE configurations depends on the fraction of exact exchange in the hybrid functional, which highlights the sensitivity of STE properties to electronic structure methods.

## Supplementary Material



## Data Availability

Structures and input files needed to reproduce the results are available on Zenodo at https://doi.org/10.5281/zenodo.15573410.

## Notes

Detailed computational parameters, convergence tests for supercell size, additional figures showing the formation and emission energies of STEs, absorption spectrum of pristine BiVO_4_, a comparison of optimal α values across different cell sizes, and a comparison of the properties of STE1 between tetragonal and monoclinic structures (PDF)
