# Supplementary material for: Competing Self-Trapped Exciton States and Multiple Emission Pathways in BiVO4
Source: J Phys Chem Lett. 2025 Jun 26;16(26):6861–5. doi: 10.1021/acs.jpclett.5c01215 (PMC12235617; doi:10.1021/acs.jpclett.5c01215)
Supplement: Supplementary file 1 [file jz5c01215_si_001.pdf]

Supporting Information: Competing Self-Trapped Exciton States and Multiple Emission Pathways in BiVO4

Tobias Möslinger, Nicklas Österbacka, and Julia Wiktor*

Department of Physics, Chalmers University of Technology, SE-412 96 Gothenburg, Sweden

E-mail: julia.wiktor@chalmers.se

Computational details

We use both CP2K[S1] and VASP[S2,S3] for DFT calculations. The former is used to determine the optimal fraction of exact exchange in the hybrid exchange as well as for structural optimization. The latter is used to determine formation energies and optical properties at higher accuracy.

CP2K

We make use of the Gaussian and Plane Waves approach to density functional theory.[S4] A double-zeta polarized MOLOPT basis set along with an auxiliary plane-wave basis expanded up to a cutoff of 800 Ry is used to expand the valence states.[S5] Core states are described using Goedecker-Teter-Hutter pseudopotentials.[S6] The PBE0-TC-LRC functional is used to describe exchange and correlation.[S7,S8] Additionally, the auxiliary density matrix method is employed to speed up calculations.[S9] Spin polarization was considered and the Brillouin zone was sampled at the gamma point. Geometry optimization was considered completed when all residual forces were below 10 meV/Å. Lattice parameters were optimized under a target external pressure of 0 bar with a pressure tolerance of 1 bar.

VASP

Final calculation of formation energies and optical transitions we performed within VASP with the hybrid PBE0(14%) functional. The plane-wave kinetic energy cutoff was set to 300 eV. PAW potentials were used, with 5 valence electrons for V and Bi and 6 for O. Spin polarization was included in the calculations for STEs. Geometries were taken directly from CP2K without further relaxation. The Brillouin zone was sampled using Γ-centered k-point meshes, adjusted for each case as indicated.

Determination of optimal α with other supercell sizes

In the main text, a supercell containing 192 atoms is used throughout, corresponding to a $2 \times 2 \times 2$ repetition of the primitive cell. This gives an optimal exact exchange fraction of $\alpha = 0.14$. To ensure that this value is sufficiently converged we determine the optimal α using $2 \times 2 \times 1$ and $3 \times 3 \times 2$ repetitions of the ideal cell as well, respectively containing 96 and 432 atoms. Both yield $\alpha = 0.14$ as shown in Fig. S1.

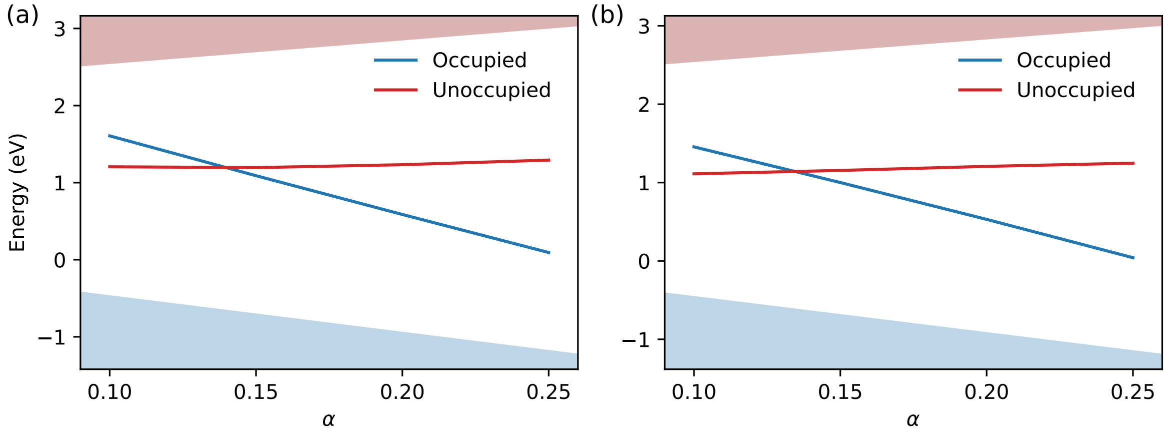

Figure S1: Optimal exact exchange fraction α determined using a) a 96-atom supercell and b) a 432-supercell. These correspond to $2 \times 2 \times 1$ and $3 \times 3 \times 2$ repetitions of the primitive cell, respectively.

Convergence of formation and emission energy with respect to the supercell size

The formation and emission energies of the self-trapped exciton (STE) are sensitive to the choice of supercell size and k-point sampling. To address this, we systematically investigate the formation energy and vertical transition in the STE geometry for varying cell sizes. The formation energy is defined as outlined in the main text. Additionally, the vertical transition energy is calculated as the energy difference between the triplet state and the ground-state singlet in the STE geometry. This difference serves as a predictor for the convergence of

emission energies expected from TD-DFT calculations.

We construct supercells containing 96, 192, 384, and 768 atoms (corresponding to 4, 8, 16, and 32 times the unit cell, respectively) by arranging the unit cell in various ways. Two types of supercells are generated: one set by straightforward multiplication of the unit cell and another by applying a transformation that rotates the unit cell by 45° in the X-Y plane. This transformation is represented by the matrix:

$$\begin{bmatrix} 1 & 1 & 0 \\ -1 & 1 & 0 \\ 0 & 0 & 1 \end{bmatrix}.$$

The specific multiplication factors for the supercells are as follows: $2 \times 2 \times 1$ for 96 atoms, $2 \times 2 \times 2$ and $\sqrt{2} \times \sqrt{2} \times 1$ for 192 atoms, $4 \times 4 \times 1$ and $\sqrt{2} \times \sqrt{2} \times 2$ for 384 atoms, and $4 \times 4 \times 2$ and $\sqrt{4} \times \sqrt{4} \times 1$ for 768 atoms. We note that some of the supercells have the same lattice parameters in the x and y direction and differ only by the length in the z direction. The structures of two different STE geometries are then inserted into each of these supercells. The energies of the neutral and triplet states are calculated by fully relaxing the structures in each case. Results are given in Fig. S2 and S3 in which we use the red color for the 'flat' cells and blue for 'elongated' cells. The reason for this distinction is that while STE2 configuration is strongly localized and can be seen as a point defect, STE1 has a distribution in the XY plane. In the plots we also include a linear extrapolation of the point to infinite S_{XY} area which is defined as the product between the corresponding lattice parameters.

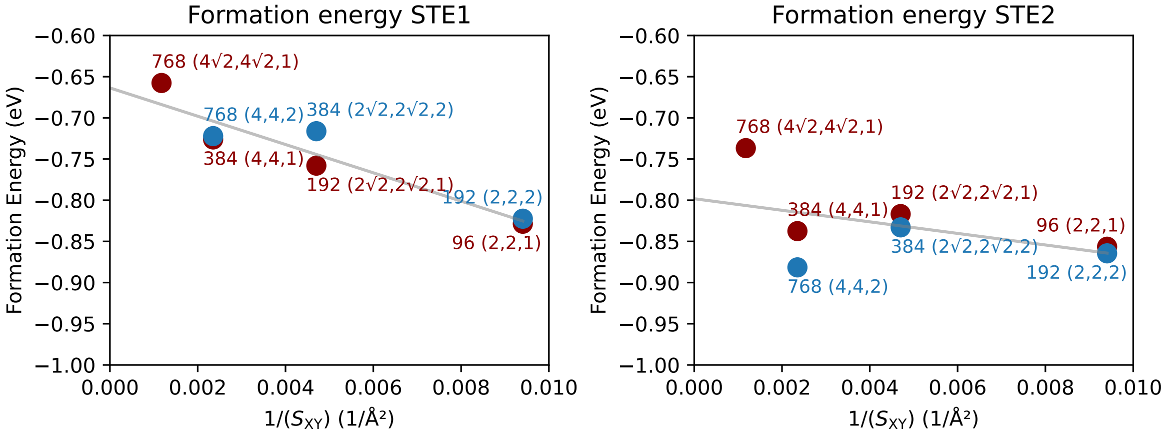

Figure S2: Formation energies plotted as a function of the inverse area $(1/S)$ for different z-dimension configurations of the cell. The red points correspond to $z = 1$ cells, and the blue points correspond to $z = 2$ cells. Linear fits are included to highlight trends.

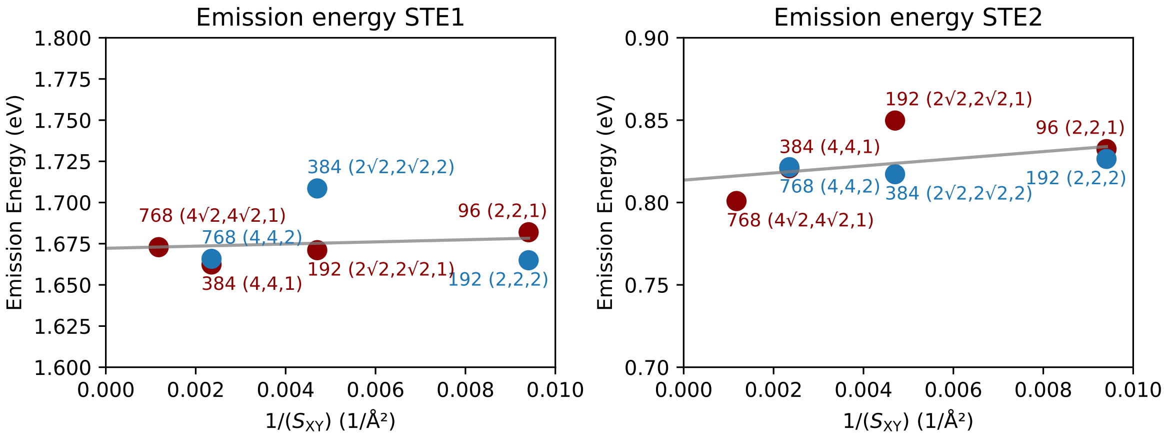

Figure S3: Emission energies plotted as a function of the inverse area $(1/S)$ for different z-dimension configurations of the cell. The red points correspond to $z = 1$ cells, and the blue points correspond to $z = 2$ cells. Linear fits are included to illustrate the relationship.

Absorption Properties of the Pristine Material

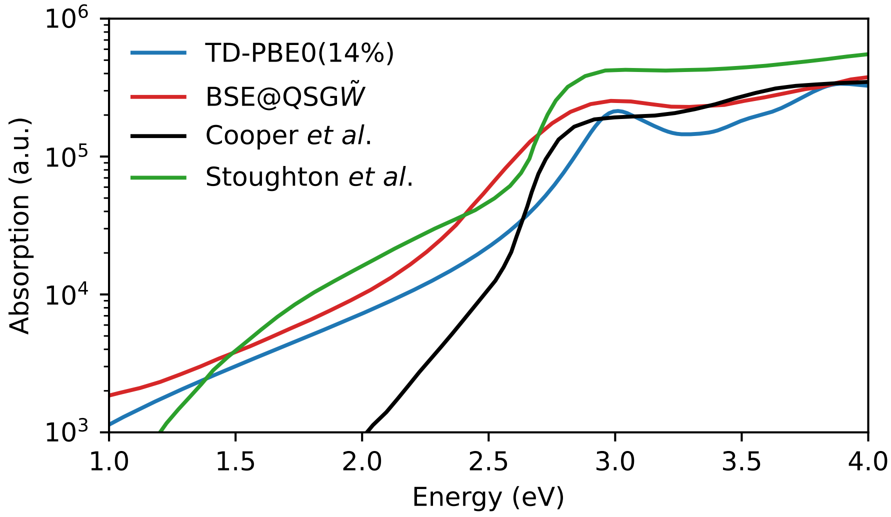

Figure S4: Absorption spectrum calculated using the TD-PBE0(14%) method, compared to BSE@QS$G\tilde{W}$ calculations and experimental data from Refs. S10 and S11.

To evaluate the accuracy of our TD-DFT approach with the PBE0(14%) hybrid functional in describing the optical properties of pristine $BiVO_4$, we compute its absorption spectrum using a 12-atom unit cell, a plane-wave cutoff energy of 300 eV, a $4 \times 4 \times 4$ k-point grid, and 20 occupied and 20 unoccupied bands.

As shown in Fig. S4, the TD-PBE0(14%) spectrum exhibits excellent agreement with previous BSE@QS$G\tilde{W}$ calculations from Ref. S12, as well as with experimental absorption spectra from Cooper *et al.*[S10] and Stoughton *et al.*[S11] While the choice of $\alpha = 14\%$ is lower than the value required to exactly match the QS$G\tilde{W}$ band gap, this is compensated by the omission of temperature effects, which are known to increase the band gap in $BiVO_4$. The resulting absorption spectrum is therefore in close agreement with both theoretical and experimental reference.

Comparison between Tetragonal and Monoclinic Phases

In this work, we consider the tetragonal scheelite structure, since it corresponds to the ground state in the computational setup used. Experimentally, however, $BiVO_4$ adopts the monoclinic structure at room temperature. We note that high fractions of exact exchange in the hybrid functional are needed to stabilize the monoclinic phase,[S13] far beyond the one determined from the Koopmans' condition. To validate our use of the tetragonal phase, we have repeated the calculations for STE1 under monoclinic symmetry: the pristine 2×2×2 supercell was relaxed in CP2K, STE1 was introduced in the triplet state and all atoms were re-optimized, and final energies were recomputed in VASP. We find the formation energy of 0.91 eV in the monoclinic structure compared to 0.90 eV in the tetragonal one. We further perform TD-DFT calculation on the two structures and compare them in Fig. S5. The transitions are almost indistinguishable, validating our choice to use the tetragonal model in the main text.

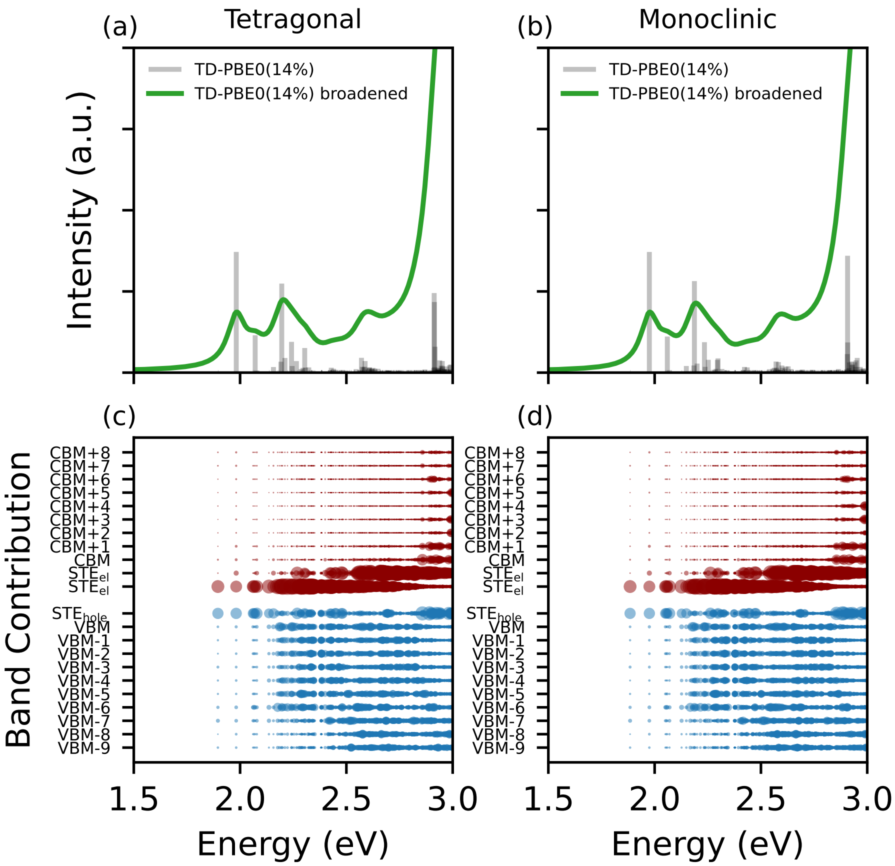

Figure S5: Optical transitions calculated for the STE1 configuration in the tetragonal (a) and monoclinic (b) structures. The broadened spectra were generated by convolution with Lorentzians with a width of 0.05 eV. (c) and (d) Band contributions to each of the transitions marked in the corresponding upper panel. For each initial (final) state, the contributions of different final (initial) states, as well as all k-points, are added up.

References

(S1) Kühne, T. D.; Iannuzzi, M.; Del Ben, M.; Rybkin, V. V.; Seewald, P.; Stein, F.; Laino, T.; Khaliullin, R. Z.; Schütt, O.; Schiffmann, F.; Golze, D.; Wilhelm, J.; Chulkov, S.; Bani-Hashemian, M. H.; Weber, V.; Borštnik, U.; Taillefumier, M.; Jakobovits, A. S.; Lazzaro, A.; Pabst, H.; Müller, T.; Schade, R.; Guidon, M.; Andermatt, S.; Holmberg, N.; Schenter, G. K.; Hehn, A.; Bussy, A.; Belleflamme, F.; Tabacchi, G.; Glöß, A.; Lass, M.; Bethune, I.; Mundy, C. J.; Plessl, C.; Watkins, M.; VandeVondele, J.; Krack, M.; Hutter, J. CP2K: An Electronic Structure and Molecular

Dynamics Software Package - Quickstep: Efficient and Accurate Electronic Structure Calculations. *J. Chem. Phys.* **2020**, *152*, 194103.

(S2) Kresse, G.; Hafner, J. Ab initio molecular dynamics for liquid metals. *Phys. Rev. B* **1993**, *47*, 558.

(S3) Kresse, G.; Furthmüller, J. Efficient iterative schemes for ab initio total-energy calculations using a plane-wave basis set. *Phys. Rev. B* **1996**, *54*, 11169.

(S4) Lippert, G.; Hutter, J.; Parrinello, M. A Hybrid Gaussian and Plane Wave Density Functional Scheme. *Mol. Phys.* **1997**, *92*, 477–487.

(S5) VandeVondele, J.; Hutter, J. Gaussian Basis Sets for Accurate Calculations on Molecular Systems in Gas and Condensed Phases. *J. Chem. Phys.* **2007**, *127*, 114105.

(S6) Goedecker, S.; Teter, M.; Hutter, J. Separable Dual-Space Gaussian Pseudopotentials. *Phys. Rev. B* **1996**, *54*, 1703–1710.

(S7) Perdew, J. P.; Ernzerhof, M.; Burke, K. Rationale for Mixing Exact Exchange with Density Functional Approximations. *J. Chem. Phys.* **1996**, *105*, 9982–9985.

(S8) Guidon, M.; Hutter, J.; VandeVondele, J. Robust Periodic Hartree-Fock Exchange for Large-Scale Simulations Using Gaussian Basis Sets. *J. Chem. Theory Comput.* **2009**, *5*, 3010–3021.

(S9) Guidon, M.; Hutter, J.; VandeVondele, J. Auxiliary Density Matrix Methods for Hartree-Fock Exchange Calculations. *J. Chem. Theory Comput.* **2010**, *6*, 2348–2364.

(S10) Cooper, J. K.; Gul, S.; Toma, F. M.; Chen, L.; Liu, Y.-S.; Guo, J.; Ager, J. W.; Yano, J.; Sharp, I. D. Indirect bandgap and optical properties of monoclinic bismuth vanadate. *The Journal of Physical Chemistry C* **2015**, *119*, 2969–2974.

(S11) Stoughton, S.; Showak, M.; Mao, Q.; Koirala, P.; Hillsberry, D.; Sallis, S.; Kourkoutis, L. F.; Nguyen, K.; Piper, L.; Tenne, D.; others Adsorption-controlled growth of $BiVO_4$ by molecular-beam epitaxy. *APL Materials* **2013**, *1*.

(S12) Wiktor, J.; Reshetnyak, I.; Ambrosio, F.; Pasquarello, A. Comprehensive modeling of the band gap and absorption spectrum of $BiVO_4$. *Physical review materials* **2017**, *1*, 022401.

(S13) Liu, T.; Zhang, X.; Guan, J.; Catlow, C. R. A.; Walsh, A.; Sokol, A. A.; Buckeridge, J. Insight into the fergusonite–scheelite phase transition of ABO4-type oxides by density functional theory: A case study of the subtleties of the ground state of $BiVO_4$. *Chemistry of Materials* **2022**, *34*, 5334–5343.
